# Inferring genetic architecture of complex traits using Bayesian integrative analysis of genome and transcriptome data

**DOI:** 10.1186/1471-2164-13-456

**Published:** 2012-09-05

**Authors:** Alireza Ehsani, Peter Sørensen, Daniel Pomp, Mark Allan, Luc Janss

**Affiliations:** 1 Department of Molecular Biology and Genetics, Faculty of Science and Technology, Aarhus University, Tjele, DK-8830, Denmark; 2 School of Medicine, University of North Carolina at Chapel Hill, Chapel Hill, NC, 27599-7264, USA; 3 Trans Ova Genetics, Sioux Center, Sioux, IA, 51250, USA

**Keywords:** Bayesian, Body Weight, Feed Intake, Genome, Transcriptome, eQTL, Variance

## Abstract

**Background:**

To understand the genetic architecture of complex traits and bridge the genotype-phenotype gap, it is useful to study intermediate -omics data, e.g. the transcriptome. The present study introduces a method for simultaneous quantification of the contributions from single nucleotide polymorphisms (SNPs) and transcript abundances in explaining phenotypic variance, using Bayesian whole-omics models. Bayesian mixed models and variable selection models were used and, based on parameter samples from the model posterior distributions, explained variances were further partitioned at the level of chromosomes and genome segments.

**Results:**

We analyzed three growth-related traits: Body Weight (BW), Feed Intake (FI), and Feed Efficiency (FE), in an F_2_ population of 440 mice. The genomic variation was covered by 1806 tag SNPs, and transcript abundances were available from 23,698 probes measured in the liver. Explained variances were computed for models using pedigree, SNPs, transcripts, and combinations of these. Comparison of these models showed that for BW, a large part of the variation explained by SNPs could be covered by the liver transcript abundances; this was less true for FI and FE. For BW, the main quantitative trait loci (QTLs) are found on chromosomes 1, 2, 9, 10, and 11, and the QTLs on 1, 9, and 10 appear to be expression Quantitative Trait Locus (eQTLs) affecting gene expression in the liver. Chromosome 9 is the case of an apparent eQTL, showing that genomic variance disappears, and that a tri-modal distribution of genomic values collapses, when gene expressions are added to the model.

**Conclusions:**

With increased availability of various -omics data, integrative approaches are promising tools for understanding the genetic architecture of complex traits. Partitioning of explained variances at the chromosome and genome-segment level clearly separated regulatory and structural genomic variation as the areas where SNP effects disappeared/remained after adding transcripts to the model. The models that include transcripts explained more phenotypic variance and were better at predicting phenotypes than a model using SNPs alone. The predictions from these Bayesian models are generally unbiased, validating the estimates of explained variances.

## Background

Large amounts of genomic information generated from Single Nucleotide Polymorphism (SNP) microarrays have become available in recent years for many species [1-3]. This genomic information is used to detect polymorphisms that contribute to variation in economically important traits, such as production traits in farm animals [3]. Microarray technology is also used to screen the expression levels of thousands of genes, i.e., the transcriptome [4,5]. Studies have shown that genetic background can have a large impact on differential expression [6]. Integrating genome and transcriptome information can help to elucidate the underlying biology of the genotype-phenotype map, using expression Quantitative Trait Locus (eQTL) mapping [7].

However, in the eQTL approach, associations between SNPs, transcript level, and phenotypes are analyzed individually. This is likely to lead to “missing heritability” [8], because corrections for multiple testing lead to a high false negative rate and multiple SNPs and transcript level that jointly explain the phenotype are ignored [9,10]. Here we propose and demonstrate Bayesian models that model all SNPs and transcript level simultaneously to obtain explained variances by the whole genome and whole transcriptome. In these models, we identify eQTLs as those SNPs whose effects disappear when transcript level are added to the model. Genomic- and transcriptomic-explained variances are further partitioned by chromosome and genome sections to offer a view of the genetic architecture on different aggregation levels.

The choice of Bayesian variable selection (BVS) models was due to its features to separate markers with large/moderate or small effects, and to locate the important regions in the genome or transcriptome which serves a better QTL mapping method because it produces clearer signals for QTL [11]. Furthermore the prediction based on genomic variables using BVS is more accurate even when the prior is not correct [11-14]. It is important to say that simpler methods suffer from “missing heritability” too [15,16].

The aim of this study was to explore the contributions of various sources of variation, such as population structure, SNP variants, and gene expression levels, to a set of growth related traits (body weight, feed intake, and feed efficiency) in mice. These traits are very important, both in terms of agricultural production and for obesity in humans. Bayesian mixed models and Bayesian variable selection models were applied to model pedigree, SNPs and/or gene expressions and to derive explained variances for these components. In addition, they were used to partition of SNPs and gene expression by chromosome and genome sections. To validate the estimates of explained variances, the predictive ability of these models was studied using cross validation.

### Data

An M16 × ICR F_2_ population of 440 mice was available with complete records for body weight at 8 weeks (BW) and 337 records for feed intake (FI) and feed efficiency (FE), measured during the period 3 weeks to 8 weeks [17]. An additional 89 pedigree records were available that described the family structure up to the F_0_ founder lines. Data was obtained in three batches and the sex of the animals was recorded. At the end of the experiment, the mice were sacrificed and liver tissue was extracted for genome-wide expression profiling. RNA isolation, cDNA synthesis, array hybridization, normalization of probe level intensity, and annotation of data were performed as described in detail by [18]. Genotypes for 1806 highly informative single nucleotide polymorphisms (SNPs) were available for each animal. These tag-SNPs were used to trace the genomic variation in this F_2_ population. Density functions of phenotypes are available in Additional file 1 and the whole data were made publicly available at ( http://gbi.agrsci.dk/~pso/BIG_genome_transcriptome/).

## Methods

The most complete model used describes phenotypes **y** (BW, FI, or FE) by an intercept μ, environmental effects of batch and sex **b**, a polygenic effect based on pedigree **u**, regressions on SNP covariates **a**, regressions on gene expression covariates **g**, and a model residual **e**, as:

(1) y=1µ+Xb+Zu+Wa+Qg+e

where **X** is the design matrix for batch and sex effects, **Z** is a design matrix that links polygenic effects to the observed records, **W** is a matrix with 1806 SNP covariates, and **Q** is a matrix with 23,698 gene expression covariates. The SNP and gene expression covariates were centered and scaled to unit variance.

Based on work of [19-22], the Bayesian mixed model version assigns normal prior to the vectors **u**, **a**, **g**, and **e** in (1), i.e., $u∼N\left(0,A\sigma_{u}^{2}\right),a∼N\left(0,I\sigma_{s}^{2}\right),g∼N\left(0,I\sigma_{g}^{2}\right),e∼N\left(0,I\sigma_{s}^{2}\right)$, where $\sigma_{e}^{2}$ is the polygenic variance and **A** is the numerator relationship matrix based on pedigree information, $\sigma_{s}^{2}$ is the per-SNP explained variance, $\sigma_{g}^{2}$ is the per-gene expression explained variance, and $\sigma_{e}^{2}$ is the residual or environmental variance. These four variances are estimated in the model using flat prior distributions, i.e., $\sigma_{u}^{2},\sigma_{s}^{2},\;\sigma_{g}^{2},\;\sigma_{e}^{2}∼\;\text{Bern}$. The remaining parameters in (1), μ and **b**, are assigned flat prior distributions, which is the Bayesian analog of fitting “fixed effects” (unshrunken) estimates. A Markov chain Monte Carlo (MCMC) algorithm was applied in the software bayz [23] to obtain samples from the posterior distribution of the model parameters $f\left(\mu ,b,u,a,g,\sigma_{u}^{2},\sigma_{s}^{2},\sigma_{g}^{2},\sigma_{e}^{2}|y\right)$. MCMC algorithms for sampling effects and variances in mixed models have been extensively described, for a general overview see [24]. The Monte Carlo accuracy of the MCMC algorithm was evaluated by correlating repeated estimates for the parameter vectors **u**, **a** and **g**, requiring a correlation >0.999 from repeated MCMC runs, and by computing the effective sample sizes for the variance components using the R Coda package [25].

The explained variance in **y** from (1) is var(**Zu**) + var(**Wa**) + var(**Qg**) + var(**e**). To obtain posterior means (PMs) and posterior standard deviations (PSDs) on the explained variances for SNPs and gene expressions, var(**Wa**) and var(**Qg**) were evaluated based on the posterior samples for **a** and **g** from the MCMC, i.e., as the PM and PSD of var(Wa^t^) values over MCMC cycles, where a^t^ is the posterior sample for **a** from MCMC cycle *t*. This procedure is not required for the polygenic variance, because **Z** is a design matrix, unlike **W** and **Q**, which are covariate matrices.

The second model used was a Bayesian variable selection model, where the approach of George and McCulloch [26] was followed to fit mixture distributions with small and large variances as the prior distribution for regression coefficients. In model (1), such a mixture prior was applied to SNPs as well as gene expression regression coefficients, with independent parameters and mixture indicators for SNPs and for gene expressions. The basic model of George and McCulloch [26] was further extended to incorporate the variances in the mixture distribution as unknown model parameters, which allows the model to learn the relative importance of SNPs and gene expressions from the data. This variable selection model thus takes the prior distributions for **a** and **g** as follows:

(2) $$a_{i}\;∼\;\gamma_{\mathrm{ai}}N\left(0,\;\tau_{a1}^{2}\right)\;+\;\left(1\;-\;\gamma_{\mathrm{ai}}\right)N\left(0,\;\tau_{a0}^{2}\right)$$

(3) $$g_{i}\;∼\;\gamma_{\mathrm{gi}}N\left(0,\;\tau_{g1}^{2}\right)\;+\;\left(1\;-\;\gamma_{\mathrm{gi}}\right)N\left(0,\;\tau_{g0}^{2}\right)$$

where $\tau_{a1}^{2}$ and $\tau_{a0}^{2}$ are the “large” and “small” variances in the mixture distribution for **a**, $\tau_{g1}^{2}$ and $\tau_{g0}^{2}$ are the “large” and “small” variances in the mixture distribution for **g**, and $\gamma_{a}$ and $\gamma_{g}$ are vectors of 0/1 indicator variables for **a** and **g**, respectively, indicating whether the *i*th element in **a** or **g**, respectively, comes from the distribution with large or small variance. The variances $\tau_{a1}^{2},\;\tau_{a0}^{2},\;\tau_{g1}^{2},\;\tau_{g0}^{2}$ were all estimated from the data using unbounded flat prior distributions. The constraints $\tau_{a1}^{2}\;>\;\tau_{a0}^{2}$ and $\tau_{g1}^{2}\;>\;\tau_{g0}^{2}$ were applied using a rejection sampler, so that “large” and “small” effects remained identifiable. The priors for the indicator variables were taken as $\gamma_{\mathrm{ai}}\;∼\;\text{Bern}\left(\pi_{a}\right)$ and $\gamma_{\mathrm{gi}}\;∼\;\text{Bern}\left(\pi_{g}\right)$, where $\text{Bern}\left(\pi \right)$means a Bernoulli distribution for a 0/1 indicator with a probability π for a 1. The parameters $\pi_{a},\;\pi_{g}$were taken as known. The MCMC implementation of this model is relatively straightforward, because conditional on the indicator variables the model remains a mixed model. The updating of the mixture indicators is described in [26]. This model is also run in the software bayz [23], and the Monte Carlo accuracy was evaluated in the same way as the mixed model version.

From the posterior samples for **a** and **g** in the variable selection model, explained variances were computed and partitioned by chromosome and by genome section. The variable selection model is more suited to make such a partitioning, because unlike the mixed model version, it allows for different variance contributions per SNP. The explained variances were evaluated in the same way as for the mixed model, by evaluating var(Wa^t^) and var(Qg^t^) over MCMC cycles *t*, except that the **a** and **g** samples are obtained under the mixture model prior assumptions. The same expressions can be straightforwardly evaluated for parts of the SNPs or gene expressions to obtain explained variances per chromosome and for small windows of SNPs within chromosomes. Variance within a chromosome was computed using a 5-SNP sliding window to obtain a genomic variance profile.

It is difficult to choose an optimal windows size as it depends on extend of LD, marker density and an arbitrary cut-off for what is considered important LD. In the data analyzed here, average R^2^ between adjacent SNPs was 0.55, and average R^2^ between SNPs two apart was 0.39, which we considered sufficiently high to warrant computation of variances in a 5-SNP window. To study the relative importance of family structure, SNPs, and gene expressions, six sub models and the complete model (1) were used. These were models that use only pedigree information (PED), only SNP data (SNP), only gene expression data (GEX), SNP + GEX, PED + GEX, PED + SNP, and the complete model PED + SNP + GEX. These models always included sex and batch effects.

The predictive ability of the models was evaluated using an 11-fold cross-validation. For body weight, 440 records were divided randomly in 11 groups, each with 40 individuals. Feed intake and feed efficiency, with 337 records in total, were randomly divided in 10 groups of 30 records and one group of 37 records. The complete model, including all variance parameters, was re-estimated on each set of 10 folds and predictions were computed for the phenotypes in the remaining 11^th^ fold. All predictions from the 11-fold cross validation were collected to compute correlations between predicted and actual phenotypes, and regressions of predicted phenotypes on actual phenotypes, using the whole data set. The slope of the regression lines of predicted phenotypes on actual phenotypes are expected to be 1 if the model produces unbiased predictions, which would validate the estimates of explained variances. The University of Nebraska Institutional Animal Care and Use Committee approved all procedures and protocols.

## Results and discussion

Table 1 presents estimates of explained variances for the three traits using the seven models considered. The results in Table 1 were obtained using the Bayesian mixed model. We first discuss the models that consider genetic and genomic information, which are the PED, SNP and PED + SNP models. The PED model is the classical polygenic model, using family structure to estimate narrow sense heritability, which yielded estimates of 42%, 53%, and 58% for BW, FI, and FE, respectively. Genomic information alone (SNP model) explained less variance, i.e., 36%, 28%, and 24% for BW, FI, and FE respectively. It is a common finding that SNPs explain less variance than the classical heritability estimates [27,28], which is attributed to causal variants having lower minor allele frequency than the genotyped SNPs [15], insufficient modeling of Identity By Descent by SNPs [16], and incomplete linkage disequilibrium (LD) between causal variants and genotyped SNPs [15]. Combining pedigree and SNP data (PED + SNP model) increased the explained variance above that of using pedigree only, i.e., for BW the PED + SNP model obtained an explained variance of 59%, compared to 42% for the PED model. This phenomenon is particularly common in the analysis of an F2 population, where increased genetic variance in the F2 can be captured by SNPs, but not by pedigree. In the PED + SNP model, the part covered by pedigree decreased compared to the PED only model, showing that SNPs cover part of the family relationships [13,14,29].

**Table 1:** Explained variance in different models for Body Weight (BW), Feed Intake (FI), and Feed Efficiency (FE)

**Trait**	**Explained variances**	**PED**	**SNP**	**GEX**	**PED + SNP**	**PED + GEX**	**SNP + GEX**	**PED + SNP + GEX**
Body Weight	E	9.96(1.93) 58%	9.82(0.94) 64%	3.57(0.9) 21%	7.07(1.77) 41%	2.43(1.01) 14%	3.08(0.77) 19%	2.06(1) 12%
	P	7.26(3.42) 42%	-	-	5.04(3.15) 29%	2.45(1.41) 14%	-	2.08(1.47) 12%
	S	-	5.63(0.9) 36%	-	5.14(1.08) 30%	-	2.9(0.67) 18%	2.82(0.73) 17%
	G	-	-	13.45(1.57) 79%	-	12.37(1.56) 72%	10.29(1.6) 63%	9.93(1.44) 59%
	Total	17.22	15.45	17.02	17.25	17.25	16.27	16.89
Feed Intake	E	155.59(42) 47%	202.89(22) 72%	151.89(27) 51%	137.63(40) 42%	95.48(36) 30%	125.91(24) 43%	80.41(34) 25%
	P	174.89(82) 53%	-	-	131.88(79) 40%	99.74(57) 31%	-	89.97(53) 28%
	S	-	79.53(22) 28%	-	56.32(22) 18%	-	56.05(19) 19%	45.09(18) 14%
	G	-	-	150.24(41) 49%	-	125.33(35) 39%	111.84(33) 38%	104.9(33) 33%
	Total	330.48	282.42	302.13	325.83	320.55	293.8	320.37
Feed Efficiency (×10,000)	E	1.59(0.44) 42%	2.40(0.26) 76%	2.23(0.3) 69%	1.53(0.44) 42%	1.09(0.48) 30%	1.88(0.3) 58%	1.07(0.46) 29%
	P	2.17(0.92) 58%	-	-	1.73(0.86) 47%	1.87(0.78) 51%	-	1.61(0.77) 44%
	S	-	0.76(0.24) 24%	-	0.39(0.22) 11%	-	0.61(0.23) 19%	0.33(0.2) 9%
	G	-	-	1.01(0.34) 31%	-	0.71(0.28) 19%	0.73(0.32) 23%	0.66(0.27) 18%
	Total	3.76	3.16	3.24	3.65	3.67	3.22	3.67

Explained variances are for residuals (E), polygenic effects (P), SNPs (S), and gene expressions (G). The table shows estimates as the posterior mean with posterior standard deviation in parentheses and the proportion of explained variance as percentage of the total.

Overall, explained variances increase by adding gene expression information (GEX; data from liver), i.e., in the most complete model (PED + SNP + GEX) explained variances were 88%, 75%, and 71% for BW, FI, and FE respectively. This confirms the assumption that gene expressions can explain a larger part of phenotypic variance than genetic or genomic information, by capturing environmental, and possibly non-additive, genetic effects through the gene expressions [5,30]. Information on the genetic architecture of these traits is best judged from the relative contributions of genomic and transcriptomic data in the SNP + GEX model.

This model shows that, for these traits, the liver transcriptome contributes a larger portion of explained variance. This is most pronounced for BW, with 18% of explained variance from the genome and 63% from the liver transcriptome. Thus, in this case, the predominant model is that SNPs regulate gene expressions to exert their effect on the phenotype.

**Figure 1:**
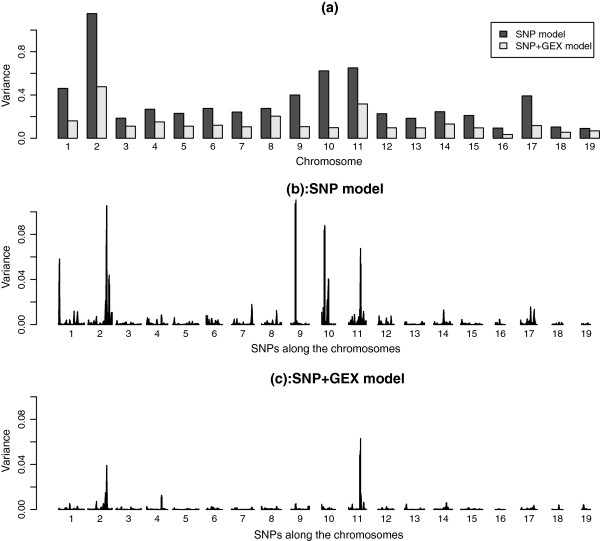
**Decomposition of the proportion of variance explained by SNPs at the level of chromosomes and individual SNPs in two models: the independent model SNP and the conditional model SNP + GEX for Body Weight.** (**a**) Explained variances from SNPs in SNP model (black) and SNP + GEX model (white) in each chromosome. (**b**) Explained variance by individual SNPs in SNP model and (**c**) SNP + GEX model.

Figure 1 shows a decomposition of the explained variances at the chromosome and sub-chromosome level for the models using genomic (SNP) and genomic with transcriptomic (SNP + GEX) data for the trait BW. For the traits FI and FE, see Additional file 2 and Additional file 3 respectively. These results are based on the Bayesian variable selection model to better differentiate between genomic regions contributing more and less variance. The genomic variances at the sub-chromosome level are explained variances in a sliding 5-SNP window. At the chromosome level, chromosome 10 particularly stands out, with a relatively large contribution from the SNPs effects via transcriptome, but only a small contribution from the genome alone in explaining the phenotype. This does not mean there is no important QTL on this chromosome. In fact, there is a large QTL on chromosome 10; however, it is an eQTL whose effect can be captured by gene expressions. Figures 1b and c show the details at the sub-chromosome level, with Figure 1b showing the explained genomic variances when fitting SNPs alone (SNP model), and Figure 1c showing the explained genomic variances when adding gene expressions to the model (SNP + GEX model). The differences between these two graphs show locations of QTLs that regulate the liver transcripts and QTLs that exert their effect on the phenotype through another route. For BW, the main QTLs are found on chromosomes 1, 2, 9, 10, and 11, and the QTLs on 1, 9, and 10 appear to be eQTLs affecting gene expression in the liver. The QTL on chromosome 2 is an intermediate case whose effect is reduced, but does not completely disappear, when adding gene expressions to the model. Thus, this chromosome 2 QTL regulates liver transcripts, but must also have effects on BW through other routes, possibly by regulating genes outside the liver. The chromosome 11 QTL is a clear case of a QTL whose effect on BW does not work via the regulation of liver transcripts. The QTL locations are in agreement with QTLs detected for body weight in other studies [17,31-33]. The same graphs for traits FI and FE are provided as supplementary material. These traits show relatively more cases where QTL effects remain after adding liver transcriptome data, which is in agreement with results in Table 1.

This method/approach is suitable for gene-level resolution. However, gene-level resolution is highly data dependent, i.e. it requires high marker density and a study population with LD blocks that span small genomic regions. In this work we have used F2 crosses from outbred lines, which has large LD blocks and this kind of data has limited resolution for fine-mapping of QTL.

One may argue that the most complete model is more interesting to investigate genetic architecture and chromosomal/sub-chromosomal variance but as we have shown SNPs and pedigree are largely confounded and they explain about the same variance. This confounded explained variance is getting worse in the case that both Pedigree and SNPs are in one model (PED + SNP model) which is shown in higher confidence intervals of explained variance by pedigree. The model with only omics information (SNP + GEX) is therefore simpler, more accurate and as effective as the model that also uses pedigree information. This is interesting for future applications of omics technologies, because we expect that pedigree information often will be absent.

**Figure 2:**
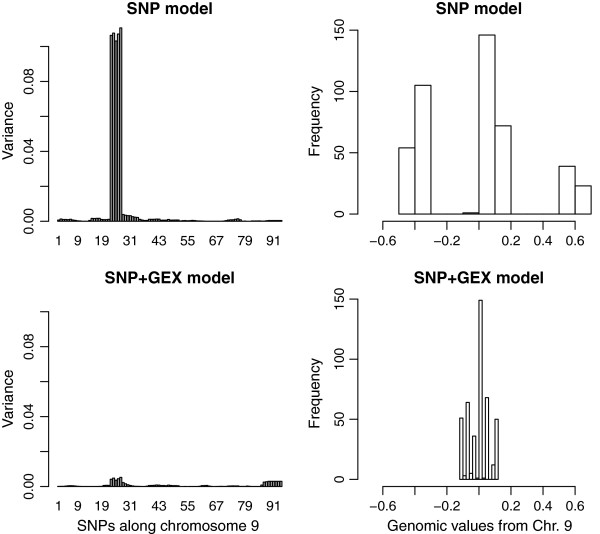
**Map of chromosome 9 for Body Weight, which follows pattern 1 such that the SNPs variance disappears when gene expression is added to the model (left).** Distribution of the genetic values in population based on chr. 9 in the SNP and SNP + GEX models (right).

**Figure 3:**
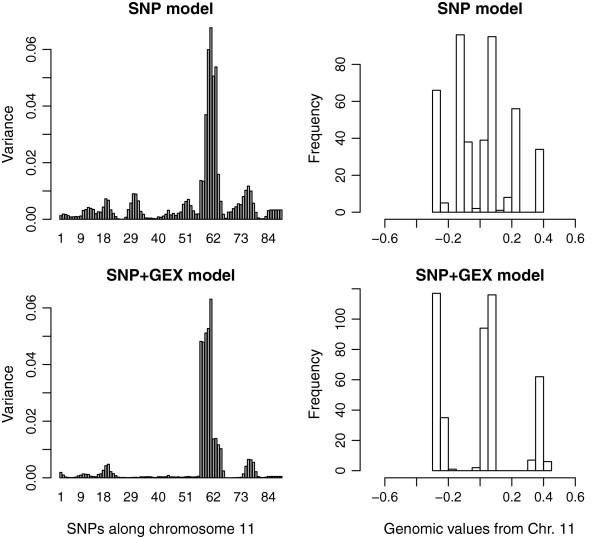
**Map of chromosome 11 for Body Weight, which follows pattern 2 such that the SNPs variance remain unchanged when gene expression is added to the model (left).** Distribution of the genetic values in population based on chr. 11 in the SNP and SNP + GEX models (right).

Figures 2 and 3 present detailed graphs of the genomic variances (left panels) and the distribution of chromosomal genomic values or breeding values [34] of the animals (right panels) for chromosomes 9 and 11, and for models fitting SNP only (top) or SNP + GEX (bottom). Breeding value is defined as the value of an individual as a parent based on sum of its genes effects [34]. Chromosome 9 is the case of an apparent eQTL, showing that genomic variance disappears, and that a tri-modal distribution of genomic values collapses, when gene expressions are added to the model. Chromosome 11 is the case of a QTL that does not regulate liver transcripts. The detailed picture of chromosome 11 shows that adding gene expressions to the model makes the effects of this QTL clearer: genomic variances outside the QTL region reduce, and a clear tri-modal distribution of chromosomal genomic values is seen in the SNP + GEX model, but not in the SNP-only model. Table 2 shows the rank correlations between the predicted values from using pedigree (PED), genomic (SNP), or transcriptomic (GEX) information. Pedigree and genomic values correlate better than pedigree/genomic values with transcriptomic values. This confirms that pedigree and genomic information overlap to a reasonable degree, but this is less true for transcriptomic information.

**Table 2:** Rank correlation (Spearman) between individual values predicted from different sources of information pedigree (PED), SNPs markers (SNP), and gene expression signals (GEX) in three traits

	**PED & SNP**	**SNP & GEX**	**PED & GEX**
**BW**	0.94	0.87	0.87
**FI**	0.93	0.87	0.88
**FE**	0.89	0.68	0.68

The prediction of phenotypes from these models, using cross-validation, is shown in Table 3, showing correlation between predicted and actual phenotypes, and the regression of predicted phenotype on actual phenotype. The scatter plot of predicted versus actual phenotypes is shown in Additional file 4. The results for explained variance and for prediction do not necessarily coincide, because prediction is also affected by the accuracy of the parameter estimates. The results show that predictions from the SNP model are all as good, or better, than from the PED model, while the explained variances from the SNP model were lower (Table 1). This can be explained by the SNP predictions being more accurate than PED predictions. Models including gene expressions show the highest correlations with phenotypes, meaning that models including gene expressions also provide accurate predictions. The regressions of predicted phenotype on actual phenotype are mostly around 1, indicating that the predictions are unbiased and that the explained variances where correctly assessed.

**Table 3:** Correlation between predicted and actual phenotypes with different sources of information

**Trait**	**Parameter**	**PED**	**SNP**	**GEX**	**SNP + PED**	**GEX + PED**	**SNP + GEX**	**SNP + GEX + PED**
Body Weight	ρ	0.76	0.8	0.87	0.80	0.87	0.88	0.88
	β	0.99	0.99	1.01	0.99	1.01	1.02	1.02
Feed Intake	ρ	0.63	0.64	0.67	0.64	0.66	0.69	0.68
	β	0.98	0.99	0.99	0.96	0.95	0.98	0.96
Feed Efficiency	ρ	0.46	0.45	0.51	0.46	0.54	0.51	0.55
	β	0.94	0.96	0.86	0.92	0.98	1	0.96

ρ Correlation between true phenotype and predicted value. β, Regression of predicted values on true phenotypes.

## Conclusions

With increased availability of various -omics data, integrative approaches are promising tools for understanding the genetic architecture of complex traits. We have developed a complementary approach to the univariate “eQTL” mapping, by considering Bayesian models that fit all genome-wide SNPs and transcript abundances in one model, and that estimate and partition explained variances by chromosome and genome segments. Our results show that, using gene expressions, more of the phenotypic variance can be explained and phenotypes can be better predicted. Predictions were also shown to be unbiased, which validates the assessed explained variances. The improvement of phenotype predictions using gene expression data will be useful for several applications in agriculture and medicine, although it should be assessed on a case-by-case basis as to whether a suitable tissue can be sampled for the gene expression measurements. Partitioning of the explained genomic variance at the level of chromosomes and genome segments showed clear examples of eQTL locations as regions where genomic variance disappears when gene expressions are added to the model. Our study used only gene expressions from the liver, and an obvious further extension is to include expressions from other tissues. The QTLs that did not disappear when transcripts are added to the model may be eQTLs that affect gene expression in a tissue other than liver. The Bayesian model is quite efficient for handling large sets of covariates, and extensions to include multiple sets of expressions will be feasible. We have not provided formal statistical tests in this model, but the Bayesian approach lends itself naturally to obtaining confidence intervals for (differences between) parameter estimates. The estimates of total explained variances from the Bayesian mixed model can also be obtained by a residual maximum likelihood (REML) approach. We verified this, and the Bayesian and REML estimates generally agree. However, using REML it is not feasible to utilize mixture priors to better discriminate between SNPs which contribute more or less variance, and to partition the variances at the sub-chromosome level, which is all straightforward in a Bayesian approach.

Our approach can easily allow up scaling to higher-density arrays, even to whole-genome sequence data with the variance components analysis as it was for gene expression probes in this study.

## Abbreviations

BW: Body Weight; FI: Feed Intake; FE: Feed Efficiency; SNPs: Single Nucleotide Polymorphisms; REML: Restricted maximum Likelihood; QTL: Quantitative trait loci; eQTL: Expression Quantitative trait loci.

## Competing interests

The authors declare that they have no competing interests.

## Authors’ contributions

AE developed the data analysis pipeline, performed statistical analyses, interpreted the results and wrote the manuscript. PS and LJ were involved in project design, statistical analyses, interpretation of results and manuscript editing. DP and MA prepared the data for the analysis. All authors have read and approved the final manuscript.

## Supplementary Material

Additional file 1 **Figure S3. **Distribution of phenotypes of traits Body Weight including 440 animals, Feed Intake and Feed Efficiency including 337 animals each. Click here for file

Additional file 2 **Figure S1. **Decomposition of the proportion of variance explained by SNPs at the level of chromosomes and individual SNPs in two models: the independent model SNP and the conditional model SNP+GEX for Feed Intake. (a) explained variances from SNPs in SNP model (black) and SNP+GEX model (white) in each chromosome. (b) explained variance by individual SNPs in SNP model and (c) SNP+GEX model. Click here for file

Additional file 3 **Figure S2. **Decomposition of the proportion of variance explained by SNPs at the level of chromosomes and individual SNPs in two models: the independent model SNP and the conditional model SNP+GEX for Feed Efficiency. (a) explained variances from SNPs in SNP model (black) and SNP+GEX model (white) in each chromosome. (b) explained variance by individual SNPs in SNP model and (c) SNP+GEX model. Click here for file

Additional file 4 **Figure S4. **Comparison of predicted breeding values versus phenotypes in the models using pedigree information only (PED), SNPs information only (SNP) and gene expression information only (GEX) for three traits Body Weight, Feed Intake and Feed Efficiency according to correlation shown in Table 3. Click here for file
