# Supplementary material for: Inferring genetic architecture of complex traits using Bayesian integrative analysis of genome and transcriptome data
Source: BMC Genomics. 2012 Sep 5;13:456. doi: 10.1186/1471-2164-13-456 (PMC3543188; doi:10.1186/1471-2164-13-456)
Supplement: Additional file 1 — Figure S3. Distribution of phenotypes of traits Body Weight including 440 animals, Feed Intake and Feed Efficiency including 337 animals each. [file 1471-2164-13-456-S1.docx]

**Supplementary Figures**

Below are the supplementary figures of genomic and transcriptomic variance decomposition for the traits Feed Intake and Feed efficiency. These figures are equivalent to figure 1 in the paper that is for Body Weight.

Supplementary Figure 1. Decomposition of the proportion of variance explained by SNPs at the level of chromosomes and individual SNPs in two models: the independent model SNP and the conditional model SNP+GEX for Feed Intake. (a) explained variances from SNPs in SNP model (black) and SNP+GEX model (white) in each chromosome. (b) explained variance by individual SNPs in SNP model and (c) SNP+GEX model.

Supplementary Figure 2. Decomposition of the proportion of variance explained by SNPs at the level of chromosomes and individual SNPs in two models: the independent model SNP and the conditional model SNP+GEX for Feed Efficiency. (a) explained variances from SNPs in SNP model (black) and SNP+GEX model (white) in each chromosome. (b) explained variance by individual SNPs in SNP model and (c) SNP+GEX model.

Supplementary Figure 3. Distribution of phenotypes of traits Body Weight including 440 animals, Feed Intake and Feed Efficiency including 337 animals each.

Supplementary Figure 4. Comparison of predicted breeding values versus phenotypes in the models using pedigree information only (PED), SNPs information only (SNP) and gene expression information only (GEX) for three traits Body Weight, Feed Intake and Feed Efficiency according to correlation shown in table 3.
