# Supplementary figures and images for: Inferring genetic architecture of complex traits using Bayesian integrative analysis of genome and transcriptome data
Source: BMC Genomics. 2012 Sep 5;13:456. doi: 10.1186/1471-2164-13-456 (PMC3543188; doi:10.1186/1471-2164-13-456)

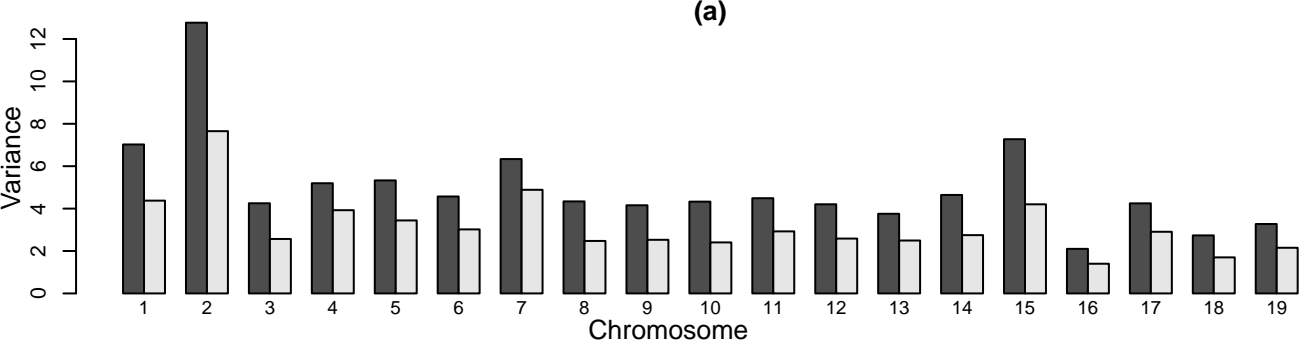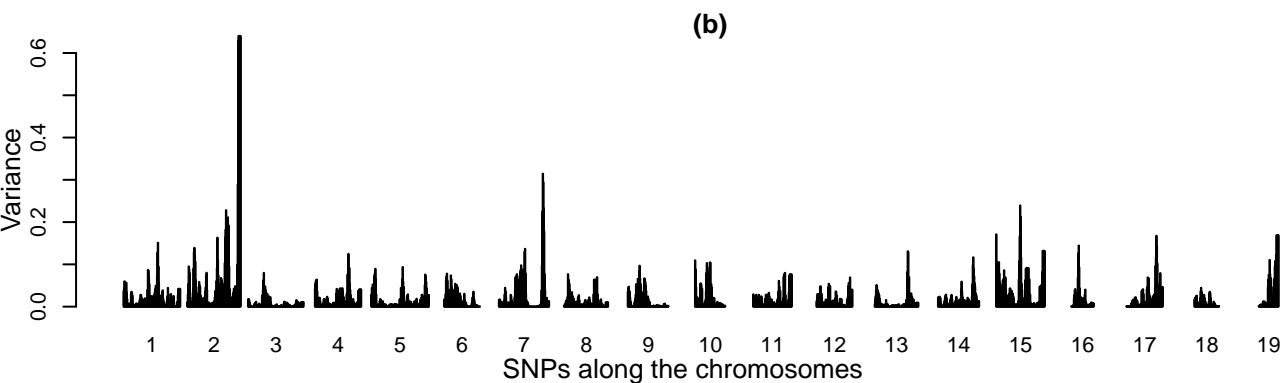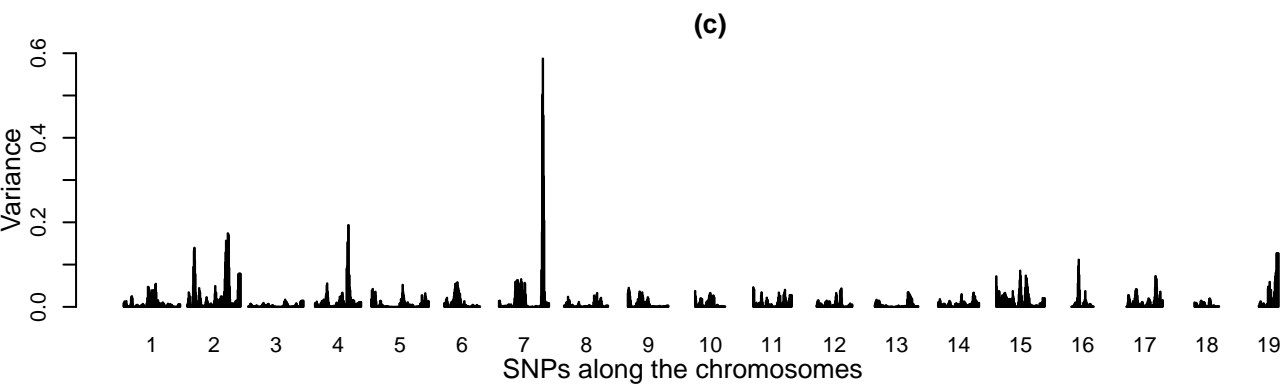

Supplement: Additional file 2 — Figure S1. Decomposition of the proportion of variance explained by SNPs at the level of chromosomes and individual SNPs in two models: the independent model SNP and the conditional model SNP+GEX for Feed Intake. (a) explained variances from SNPs in SNP model (black) and SNP+GEX model (white) in each chromosome. (b) explained variance by individual SNPs in SNP model and (c) SNP+GEX model. [file 1471-2164-13-456-S2.pdf]

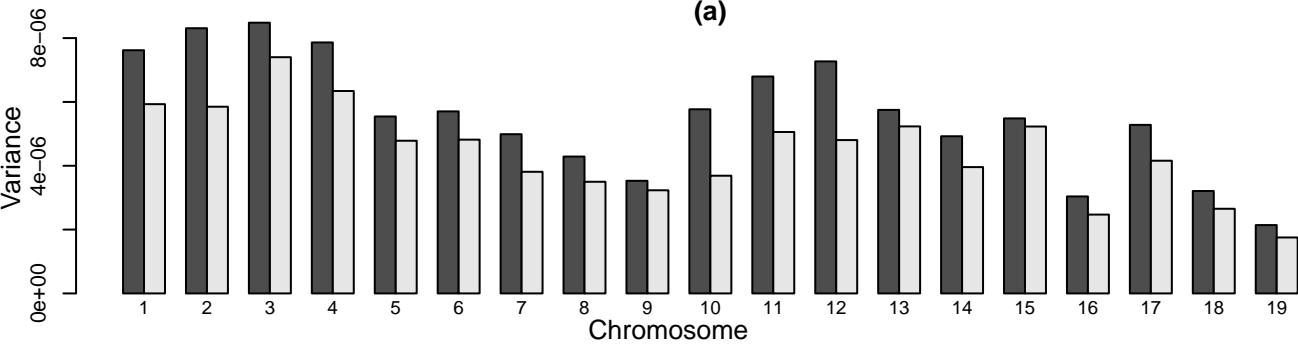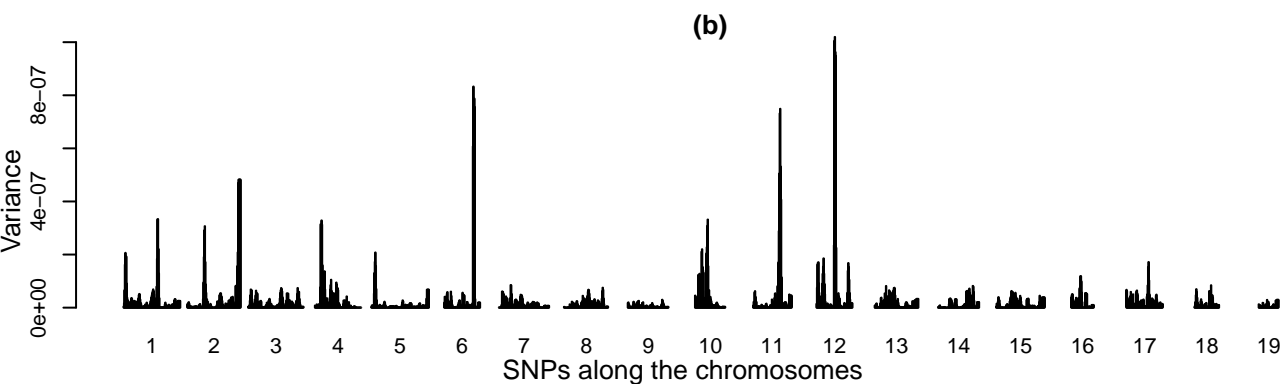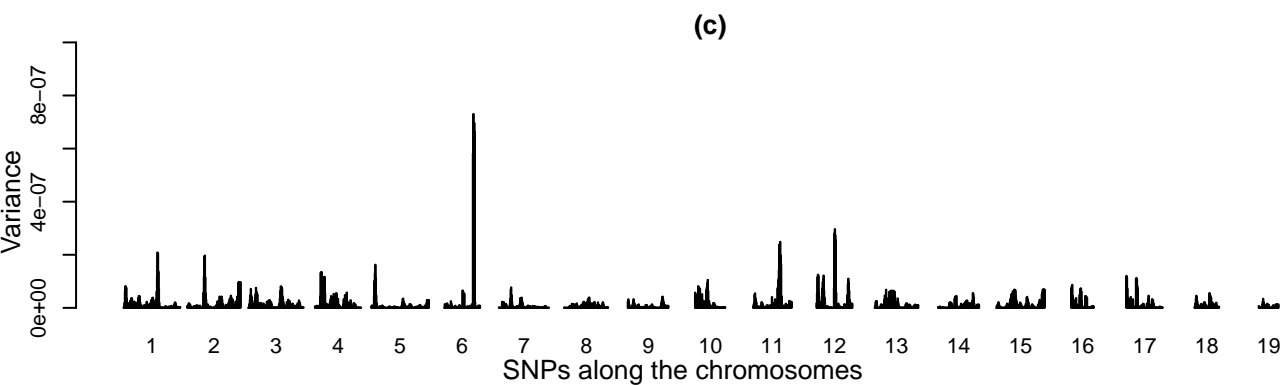

Supplement: Additional file 3 — Figure S2. Decomposition of the proportion of variance explained by SNPs at the level of chromosomes and individual SNPs in two models: the independent model SNP and the conditional model SNP+GEX for Feed Efficiency. (a) explained variances from SNPs in SNP model (black) and SNP+GEX model (white) in each chromosome. (b) explained variance by individual SNPs in SNP model and (c) SNP+GEX model. [file 1471-2164-13-456-S3.pdf]

**Body Weight**

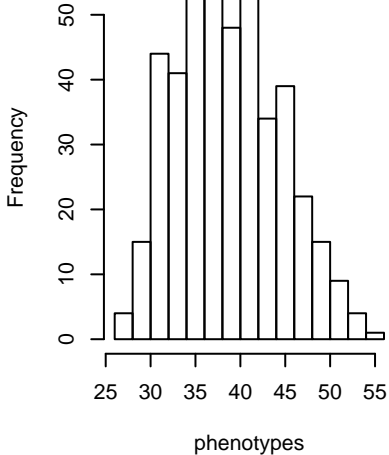

**Feed Intake**

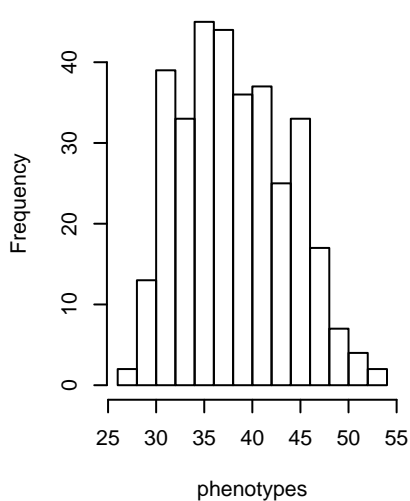

**Feed Efficiency**

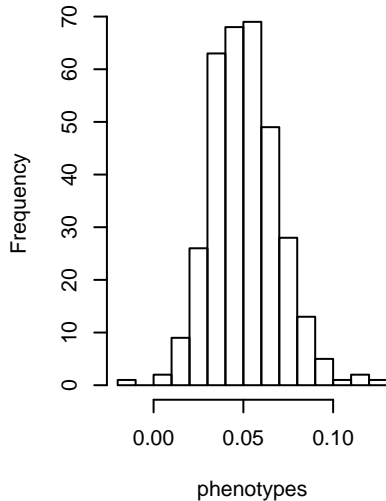

Supplement: Additional file 4 — Figure S4. Comparison of predicted breeding values versus phenotypes in the models using pedigree information only (PED), SNPs information only (SNP) and gene expression information only (GEX) for three traits Body Weight, Feed Intake and Feed Efficiency according to correlation shown in Table 3. [file 1471-2164-13-456-S4.pdf]
